# Identification of Kinase Inhibitors that Target Transcription Initiation by RNA Polymerase II

**DOI:** 10.18632/oncotarget.212

**Published:** 2011-01-27

**Authors:** José M. Morachis, Ruo Huang, Beverly M. Emerson

**Affiliations:** ^1^ Regulatory Biology Laboratory, The Salk Institute for Biological Studies, La Jolla, CA, USA; ^2^ Pharmaceutical Sciences, University of California, San Diego, La Jolla, California, USA

**Keywords:** RNA polymerase II, transcription therapy, core promoters, kinase inhibitors, p53, p21, Fas/APO1

## Abstract

Our current understanding of eukaryotic transcription has greatly benefited from use of small molecule inhibitors that have delineated multiple regulatory steps in site-specific initiation and elongation of RNA synthesis by multiple forms of RNA polymerase (RNAP). This class of “transcription” drugs is also of therapeutic interest and under evaluation in clinical trials. However, to date very few small molecules that directly abolish transcription have been identified, particularly those that act at the level of RNAP II initiation. Using a biochemical assay that measures transcription from recombinant, natural p53-responsive promoters and an artificial “super” promoter, we have identified three distinct small molecules that inhibit mRNA synthesis in vitro. Unexpectedly, these are kinase inhibitors, Hypericin, Rottlerin, and SP600125, with known substrates, which we find also strongly impair transcriptional initiation (IC50s = μM range) by targeting specific components of the RNAP II pre-initiation complex. When measured before and during transcription in vitro, one common target of inhibition by all three compounds is modification of the TATA Binding Protein (TBP) within the RNAP II holocomplex as it converts to an active transcribing enzyme. On this basis, by blocking the critical step of TBP modification, transcriptional initiation is effectively abolished even on structurally distinct core promoters.

## INTRODUCTION

Eukaryotic RNA polymerase II (RNAP II) and basal transcription factors, such as TFIID, TFIIH, TFIIB, assemble into versatile pre-initiation complexes (PIC) on core promoters to regulate initiation of mRNA synthesis. Core promoters extend from ~-50 to + 50 bp relative to the start site of transcription at +1 and are composed of different combinations of specific DNA sequence motifs such as the TATA box, initiator (INR), TFIIB recognition element (BRE), downstream promoter element (DPE), and motif ten element (MTE). The exact combination of these DNA motifs can differentially modulate the assembly of PIC complexes, start site precision of initiation, and transcriptional kinetics from specific promoters [1, 2]. After PIC assembly, multiple enzymatic processes must take place for productive mRNA synthesis to occur at the sequential steps of initiation, elongation, and RNA processing. Because transcription is dependent on diverse enzymatic reactions such as phosphorylation, ubiquitinylation, and acetylation, it is possible to inhibit mRNA production using specific pharmacological agents. For example, the transition from RNAP II pre-initiation to initiation and finally elongation is influenced by modifications within its carboxyl-terminal domain (CTD), which then functions as a platform for the ordered assembly of different pre-mRNA processing machinery [3]. Inhibition of cyclin-dependent kinases (Cdk) Cdk7 and Cdk9 abolishes RNAP II phosphorylation at specific serine residues in the CTD and prevents transcriptional elongation [4]. DRB (5,6-dichloro-1-b-D-ribofuranosylbenzimidazole), which specifically inhibits CTD phosphorylation at serine 2, has been a particularly useful reagent in the transcription field because it has facilitated the dissection of multiple elongation steps and led to the identification of important transcription factors [5]. Advanced analogs of DRB such as Flavopiridol have also been developed and have shown promising therapeutic potential for several types of human cancer [6]. Known Cdk inhibitors act by competing for the ATP binding site on target kinases and have broad-spectrum substrate specificities, including Cdk2/cyclin E, Cdk7/cyclin H and Cdk9/cyclin T [7]. Interestingly, Flavopiridol and other drugs that affect basal transcriptional steps were initially thought to inhibit kinases involved in cell cycle progression or other signaling pathways [8-10].

It has been proposed that the transcription machinery itself may be a pivotal stress sensor that directs cell fate decisions by gauging the severity of damage and activating the p53 tumor suppressor pathway [11]. In this regard, several studies have shown that blocking global cellular mRNA synthesis in human cancer cell lines by the Cdk inhibitors Roscovitine (Seliciclib, CYC202), Flavopiridol, DRB, and H7 induces a strong stress response resulting in nuclear accumulation of p53, induction of certain p53 target genes, and apoptosis [12-14]. On this basis, selective interference of transcription has become an active area of pursuit for the development of potential anti-tumor therapeutics [13]. Indeed, Flavopiridol and UCN-01 were the first Cdk inhibitors to be tested in clinical trials and have shown promising results, particularly in treating certain chronic leukemias or in combination therapy [14-16]. With the increasing availability of commercial drug libraries, it is now possible to identify novel small molecules that promote apoptosis through transcription interference. Our aim was to use *in vitro* transcription assays to identify new transcription inhibitors that act at a defined step in mRNA synthesis, initiation. To date, very few inhibitors of eukaryotic RNA initiation have been identified, with the exception of the mushroom toxin, alpha-amanitin, a cyclic peptide that acts by binding directly to RNAP II and preventing its translocation [17].

In this study, we analyzed the impact of multiple kinase inhibitors on the activity of three recombinant DNA templates containing distinct core promoter structures: two natural p53-responsive promoters and an artificial “super” promoter using a well-characterized *in vitro* transcription assay. This enabled us to identify three compounds, Hypericin, Rottlerin, and SP600125 that are each strong inhibitors of RNA synthesis. In contrast to DRB or Flavopiridol, drugs that abolish elongation by decreasing bulk cellular levels of phosphorylated CTD serine 2 phosphorylation, these compounds specifically inhibit early steps in transcription initiation by affecting enzymatically engaged RNAP II/Promoter complexes. A shared target of all three compounds is inhibition of modification of the TATA Binding Protein (TBP) within the RNAP II holocomplex as it converts to an actively transcribing form. In addition, we observe drug-specific effects on CTD phosphorylation of both bulk cellular and promoter-bound RNAP II. This reveals an unexpected role for diverse protein kinase inhibitors in directly regulating transcriptional initiation and expands their known substrate specificities to include essential factors that function on structurally distinct core promoters.

## RESULTS

### Screening compound libraries by *in vitro* transcription

**Figure 1:**
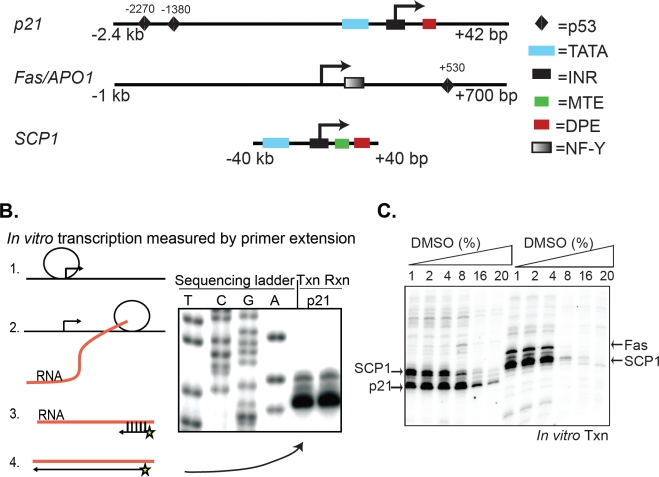
Screening compound libraries by *in vitro* transcription (A) Structures of the *p21, Fas/APO1*, and *SCP1* promoters used as *in vitro* transcription templates. Specific core promoter regulatory elements are defined in the text. (B) Diagram of the in vitro transcription assay showing: (1) Pre-initiation complex (PIC) formation and initiation of RNA synthesis, (2) RNAP II elongation and production of mRNA, (3) assay of in vitro synthesized RNA by annealing of radioactively labeled DNA primer, (4) primer extension and detection by PAGE. (C) Transcriptional analysis of *p21, Fas/APO1*, and *SCP1* as a function of increasing amounts of DMSO.

To test the ability of a library of kinase inhibitors to affect RNAP II-dependent transcription, we employed an *in vitro* assay that uses nuclear protein extracts from human tissue culture cells [18], as a source of RNAP II and transcription components. These reactions were programmed with supercoiled plasmids containing recombinant promoters that drive expression of reporter genes. This assay can distinguish between two distinct steps in transcription, initiation of RNA synthesis by RNAP II and elongation of RNA transcripts. Although several inhibitors of elongation are known (DRB, Flavopiridol) [19], very few agents that impair initiation have been identified, except a-amanitin. For this reason, we specifically measured RNAP II-dependent initiation in our assays. The recombinant DNA templates we analyzed consisted of two natural human promoters, *p21 and Fas/APO1*, and one synthetic promoter, the Super Core Promoter (*SCP1*). *p21* and *Fas/APO1* are physiologically important p53 target genes that regulate cell cycle arrest and apoptosis, respectively [20-22]. Both *p21* and *Fas/APO1* were previously characterized by *in vitro* transcription and can drive robust RNA synthesis in this assay [23]. Furthermore, *p21* and *Fas/APO1* represent two structurally distinct types of natural promoters (Figure 1A). *p21* contains multiple classic core promoter elements such as a TATA box, initiator (INR), and downstream promoter element (DPE). Whereas *Fas/APO1* lacks these canonical elements but contains a critical NF-Y response element near the +1 start site of transcription. NF-Y is a bifunctional transcription factor that regulates basal expression of Fas/APO1 *in vivo* [23]. The *SCP1* promoter is a synthetically designed chimeric promoter constructed by using sequence motifs from viral as well as cellular genes [24]. We included the *SCP1* template in all of our transcription reactions, containing either *p21* or *Fas/APO1* plasmids, as a positive internal control because of its strong activity *in vitro*. Importantly, use of these three DNA templates allowed us to screen for compounds that could inhibit the initiation of RNAP II transcription from structurally diverse promoters.

In the in vitro transcription assay, mRNA synthesis is detected by primer extension, in which purified transcripts are annealed to a short, ^32^P-labeled DNA primer followed by Reverse Transcriptase-mediated extension to generate transcripts of known length, and visualized by PAGE (Figure 1B). Due to the multiple steps involved in this assay, it is not well-suited for use in high-throughput screening of large compound libraries. However, this approach has multiple advantages that make it attractive to screen a small collection of compounds. For example, one can selectively analyze the effect of compounds or other therapeutics in targeting distinct steps in transcription, such as PIC assembly, initiation (up to 100 bp), elongation and termination. In the present study, we directly measured RNAP II-directed initiation only, which was independent of chromatin structure, nuclear localization, or other upstream processes that complicate the interpretation of cell-based assays, thus greatly simplifying our analyses. To begin, we tested the sensitivity of in vitro transcription to DMSO since drugs are commonly dissolved in this solvent. Activity from the three promoters was robust in buffers containing up to 4% DMSO (v/v) (Figure 1C), which set the limit for the volume of compound that could be added to each reaction.

### Identification of specific kinase inhibitors that directly impair transcriptional initiation

**Figure 2:**
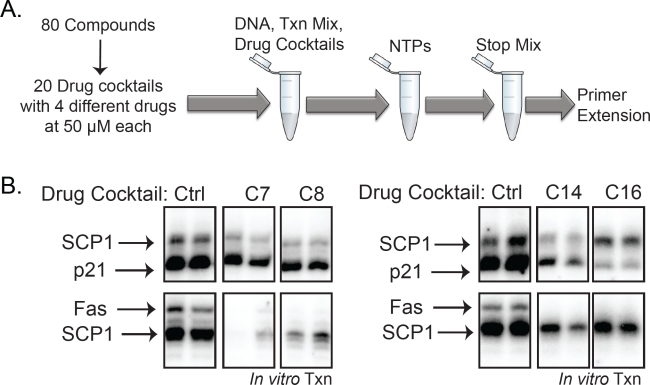
A kinase inhibitor screen identified 4 cocktails that block *in vitro* transcription (A) Diagram of the *in vitro* transcription drug screen using 80 kinase inhibitors from BIOMOL (BML-2832). The 80 compounds were mixed into 20 cocktails containing 4 drugs each at final concentrations of 50μM in the transcription reactions. (B) Four of the drug cocktails significantly reduced *in vitro* transcription of all three templates, *p21, Fas/APO1*, and *SCP1*, relative to controls.

We screened 80 commercial kinase inhibitors (BIOMOL International, see Supplemental Table 1) for their activities towards early steps in transcription on the *p21, Fas/APO1*, and *SCP1* promoters. To facilitate screening, the 80 kinase inhibitors were first combined into 20 cocktail mixes each containing four drugs. The 4-drug cocktails were added (50μM final concentration) to the transcription reactions before initiating RNA synthesis with nucleotide triphosphates (NTPs) (Figure 2A). The resulting transcripts were measured by primer extension, followed by PAGE, and compared using a phosphoimager. Using this strategy, we identified four cocktail mixes (C-7, C-8, C-14, and C-16) that significantly reduced transcriptional activity from each of the three promoters (Figure 2B).

**Figure 3:**
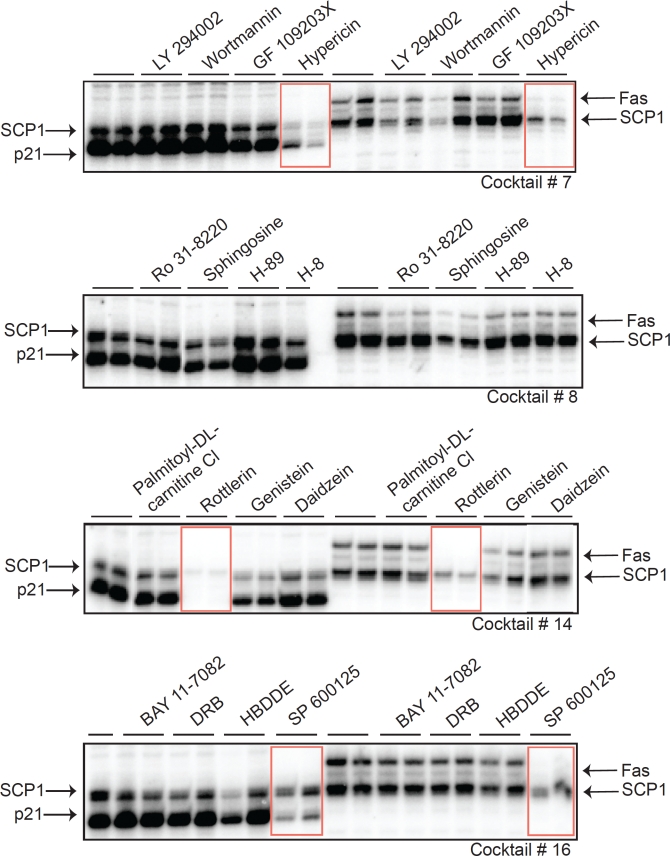
Identification of specific drugs that inhibit RNAP II-dependent initiation Three active compounds within the drug cocktails were identified that effectively inhibited transcriptional initiation and are highlighted with red rectangles. *In vitro* transcription reactions were performed in duplicates. The compounds are Hypericin (from cocktail #7), Rottlerin (from cocktail #14), and SP600125 (from cocktail #16).

The four cocktail mixes were further investigated by testing each of the compounds individually. We observed that only a single compound from cocktails C-7, C-14, and C-16 significantly inhibited transcriptional activity (Figure 3). Interestingly, none of the individual compounds in cocktail 8 were able to reduce transcription, which suggests a requirement for combinatorial inhibition in this particular mix. The three active compounds identified using this strategy were Hypericin, Rottlerin, and SP600125. These three small molecules have not previously been implicated, to our knowledge, in blocking basal transcription initiation. Hypericin induces apoptosis in cancer cells and is a potent antiviral agent [25-27]. Hypericin has also been used as a photocytotoxic compound, becoming active towards other targets upon intense light activation. This drug has been shown to inhibit protein kinase C (PKC), irreversibly damage the sarco/endoplasmic reticulum and other cellular membranes, decrease cellular pH, and inhibit mitochondrial function [27-29]. Rottlerin (mallotoxin) is considered to be a strong inhibitor of PKC delta but several studies have reported that it can inhibit other targets without affecting PKC delta [30-32]. SP600125 is a novel and selective inhibitor of c-Jun N-terminal kinase (JNK) (5). These three compounds have very different substrate targets, yet each can effectively impair RNAP II initiation on the structurally distinct promoters that we examined.

**Figure 4:**
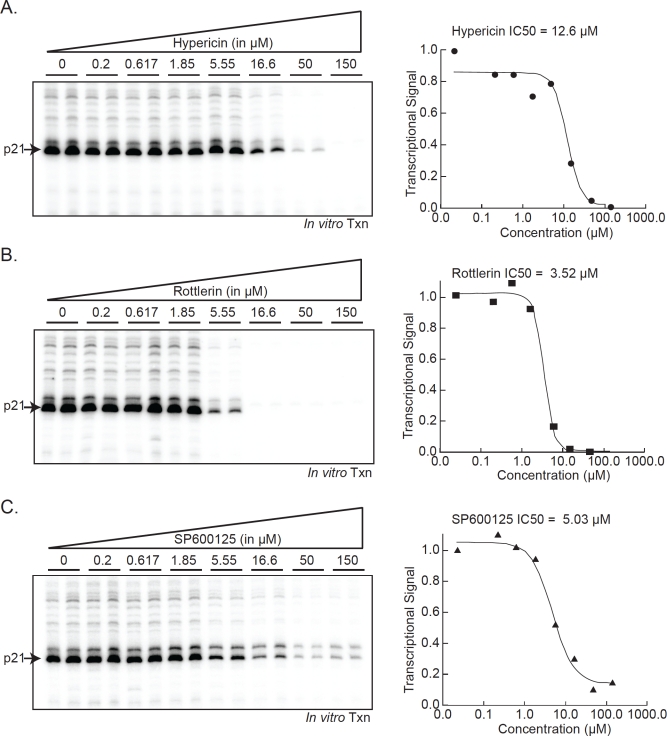
Analysis of the half-maximal inhibitory concentration (IC50) for kinase inhibitors of transcription initiation Increasing concentrations (0-160μM) of Hypericin, Rottlerin, and SP600125 were added individually to *in vitro*. transcription reactions using the *p21* promoter template. Reactions were analyzed as described and quantified using a phosphoimager.

We further characterized these compounds by analyzing their half maximal inhibitory concentrations (IC50) to measure their individual effectiveness of the drugs towards transcription, specifically using the *p21* promoter template. The approximate IC50 values were: Hypericin, 12.6μM; Rottlerin, 3.52μM; and SP600125, 5.03μM (Figure 4). Hypericin has been reported to be an effective inhibitor of epidermal growth factor with IC50 values between 0.37-8.7μM [25]. Our data demonstrate that photo-inactivated Hypericin can inhibit transcription at slightly higher concentrations. Rottlerin has been frequently used as a specific inhibitor of PKC delta and has been reported to have an IC50 of 3-6μM [31, 33]. Another study examining multiple compounds found that Rottlerin does not inhibit PKC delta but instead inhibits PRAK and MAPKAP-K2 potently, with IC50 values of 1.9μM and 5.4μM, respectively [30]. Our results suggest that Rottlerin inhibits RNAP II transcription initiation with comparable specificity as that towards its known substrates, PKC delta, MAPKAP-K2, and PRAK. SP600125, a well-characterized inhibitor of JNK, can block c-Jun phosphorylation in cells with an IC50 of 5–10 μM. However, biochemical assays using purified components produced IC50 values of 0.11μM [34]. SP600125 is an ATP competitor, thus the observed differences in IC50 values from biochemical and cell-based assays most likely reflects high ATP levels in cells. We used non-limiting amounts of ATP in the *in vitro* transcription reactions and our observed IC50 of 5.03μM probably represents a more “*in vivo*” concentration.

### Effect of specific kinase inhibitors on components of the transcription initiation complex

**Figure 5:**
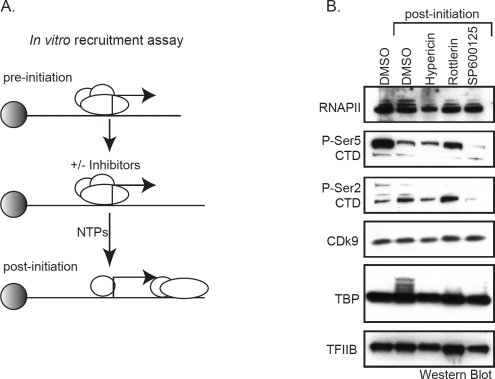
Immobilized transcription and recruitment assay (A) Diagram of the immobilized transcription and recruitment assay (see text for details). (B) HeLa nuclear extracts and *p21* promoters immobilized on magnetic beads were mixed in the presence or absence of 50μM Hypericin, Rottlerin, or SP600125. RNA synthesis was initiated with NTPs and RNAP II complexes captured during active transcription. Initiation complexes were then analyzed by Western blotting using the indicated antibodies to components of the initiation complex. In lane 1, NTPs were omitted in order to compare pre-initiation to transcriptionally active, post-initiation complexes.

Known kinase inhibitors that interfere with transcription, such as Flavopridol and DRB, typically block phosphorylation of the RNAP II C-terminal domain (CTD), which abolishes elongation. We tested Hypericin, Rottlerin, and SP600125 for their ability to inhibit serine 2 or serine 5 phosphorylation of the RNAP II-CTD in human colon cancer HCT116 cells. We found that each drug had relatively little effect on bulk, cellular RNAP II CTD phosphorylation compared to DRB (see Supplemental Data). To refine this analysis and explore how these compounds may inhibit transcription when RNAP II is actually assembled on a target promoter, we tested their individual activities towards several components of the initiation complex using immobilized DNA templates. In this assay, the transcriptional machinery in HeLa extracts is “recruited” to p21 promoter templates that are immobilized on magnetic beads, excess unbound protein is then washed from the beads leaving engaged RNAP II “pre-initiation” complexes on the promoters. Transcription is subsequently initiated by the addition of NTPs, which converts the RNAP II machinery to a “post-initiation” phase (diagrammed in Figure 5A). Capturing these functionally distinct RNAP II complexes on immobilized p21 promoters enabled us to examine the effect of each kinase inhibitor on the protein composition and modification status of several transcription components by Western blotting using appropriate antibodies. As shown in Figure 5B, nearly equal amounts of RNAP II were assembled and retained after NTP addition on promoters in the presence or absence of the each of the kinase inhibitors, with the exception of Hypericin which showed a slight decrease in bound RNAP II. Similarly, no effect of any drug was observed on TFIIB, a required initiation factor that interacts with the BRE core promoter element; or Cdk9, a kinase that is required for RNAP II elongation through phosphorylation of CTD-serine 2 and the target of inhibition by DRB and Flavopiridol [19]. Interestingly, only SP600125 demonstrated a clear inhibition of phosphorylation of promoter-bound RNAP II at both serine 2 and serine 5 within the CTD. Whether SP600125 inhibits distinct kinases that phosphorylate each residue or a single kinase that acts upstream of these events is unknown at present. One common feature of each of the three drugs is the striking loss of mobility shift of the promoter-bound TATA Binding Protein (TBP). In the absence of drug (DMSO lanes), TBP migrates as a single band when part of the pre-initiation complex (lane 1). However upon NTP addition, which activates transcription by RNAP II, bound TBP appears to undergo a series of post-translational modifications as indicated by a trailing shift to higher molecular weight species (compare lanes 1 and 2). These are most likely phosphorylated isoforms because they are inhibited by each of the three drugs. Thus, a common target of inhibition by Hypericin, Rottlerin, and SP600125 is TBP modification within the RNAP II holocomplex as it converts to an active enzyme. On this basis, by blocking the critical step of TBP conversion, transcriptional initiation is effectively abolished.

## DISCUSSION

Using an *in vitro* transcription assay, we examined the effect of 80 previously characterized kinase inhibitors specifically on the initiation of RNA synthesis by human RNAP II from three structurally diverse core promoters. This assay, although labor intensive, is unique in providing a semi-purified cell-free system in which to directly analyze drug activity on components of the transcriptional machinery. To facilitate screening the library of 80 commercially available kinase inhibitors, we initially tested drug cocktails containing combined compounds. We discovered four cocktails that demonstrated significant reduction in transcriptional activity and further characterized each compound separately. This led to the identification of three novel inhibitors of RNAP II-dependent transcriptional initiation: Hypericin, Rottlerin, and SP600125. These three compounds are thought to have alternative targets and, in fact, many kinase inhibitors are pan-specific, yet they are routinely used to block “specific” enzymes. Thus, studies using Hypericin, Rottlerin, or SP600125 and many other protein kinase inhibitors (PKIs) are likely to have pleiotrophic effects, particularly in cell-based studies. It is therefore striking that novel and unexpected targets of these broad-spectrum PKIs could be identified in such a direct and defined assay as one that measures transcriptional initiation.

We characterized the strength of inhibition towards transcription by calculating the IC50 for each compound. This revealed that the specific activity of these compounds was relatively strong. Rottlerin was the strongest inhibitor of initiation, with an IC50 of approximately 3.52μM. The compounds SP600125 and Hypericin were also effective in the micromolar range, with IC50 values of 5.03μM and 12.6μM, respectively. Previous reports using these compounds demonstrated their potential towards reducing transcription. For example, exposure of human Jurkat T-cells to Hypericin resulted in down-regulation of global mRNA synthesis, culminating in a strong, time-dependent cytotoxicity [34]. Our finding that Hypericin directly targets components of the RNAP II transcription complex to impair initiation may provide mechanistic insight into this observation. In addition, SP600125 was reported to reduce mRNA expression of the *COX-2* gene in cell-based studies, potentially by inhibiting JNK [35]. It is possible that down-regulation of COX-2 expression can be attributed to the ability of SP600125 to directly inhibit transcription complexes bound to the COX-2 promoter.

Most kinase inhibitors that block transcription usually function by preventing global cellular phosphorylation of RNAP II at serine 2 within the CTD, which affects elongation rather than initiation. Interestingly, our experiments reveal that Hypericin, Rottlerin, and SP600125 have relatively low inhibitory activity towards CTD phosphorylation of global RNAP II (Figure S1). Only by analyzing phosphorylation of promoter-bound RNAP II initiation complexes using immobilized DNA templates were we able to detect blockage of RNAP II CTD phosphorylation at both serine 2 and 5, specifically by SP600125. Using the immobilized assay, we also observed a shift in electrophoretic mobility (presumably due to protein modification) of promoter-bound TBP upon addition of nucleotide triphosphates (NTPs) to initiate transcription. Surprisingly, conversion of TBP to transcription-dependent modified isoforms was completely blocked by each of the three compounds; the TBP modification disappears and looks exactly like TBP bound before transcription initiation. A previous study demonstrated that yeast TBP could be phosphorylated by CK2, which copurifies with the TFIID holocomplex and by DNA-dependent protein kinase (DNA-PK) [36-38].

Additional studies will be required to fully understand the mechanism of action of these compounds and their relevant kinase targets. It is noteworthy that the three compounds inhibit TBP phosphorylation from architecturally diverse promoters, revealing a requirement for TBP within the TFIID complex from both TATA box-containing (*p21* and *SCP1*) and TATA-less (*Fas/APO1)* promoters. The identification of these compounds adds to the limited toolbox available to scientists studying transcription regulation. Hopefully, they will aid in finding new mechanistic information and provide additional drugs beyond those currently used to study RNAP II transcription. In addition, analogues of these compounds may provide new therapeutic candidates to treat some types of human diseases.

Many therapeutic drugs have been used to treat specific diseases without understanding their mechanism of action. In this regard, *in vitro* transcription analyses may be quite valuable, especially when adapted to a high-throughput format, since this assay is highly sensitive and has been used extensively to decipher fundamental mechanisms of transcription from core promoter elements, activators, and repressors. Moreover, this versatile assay can be modified to assess the effects of chemical and biological agents on transcription that is regulated by specific activators (i.e. p53-dependent transcription), repressors, or enzymatic complexes. In addition, any DNA template can be assembled into nucleosomal structures and used to assay the effect of compounds on such regulatory steps as protein-targeted chromatin remodeling, histone modification, or other epigenetic events [18, 39, 40]. Consequently, the effect of small molecules or biologics on gene regulation can be analyzed at a very precise mechanistic level.

## MATERIALS AND METHODS

### *In vitro* transcription assays

Nuclear protein extracts from HeLa cells were prepared as described (Dignam et al. 1983). Transcription reactions included 10μl (~5-6μg/μl) HeLa Nuclear Extract (HNE), 15μl HeLa Dialysis Buffer (HDB) (20mM Hepes-pH7.9, 50mM KCl, 1mM DTT, 0.2mM EDTA, 10% glycerol) and 25μl transcription mix (0.4mg/ml BSA, 20mM HEPES-pH 7.9, 70mM KCl, 3mM DTT, 1.2mM NTPs, 1-3mM MgCl2, 0.5μl RNase inhibitor per reaction) and 500ng supercoiled plasmid DNA templates. For the drug inhibition screen, NTPs were omitted from the transcription mix and the PIC was allowed to form for 30min at room temperature before adding 2μl of NTP mix to start the reaction (final volumes were adjusted with HDB). Transcription reactions were incubated in a 30°C water bath. Reactions were stopped and processed using reagents from Zymo Research (RNA Clean-up Kit-5) by adding 200μl of RNA binding buffer, applying the mixture to columns, washing two times with wash buffer and then eluting with 8μl of RNAse-free water.

### Primer extension analyses

Primer extension was performed by adding 3μl of primer annealing mix (10mM Tris, 1mM EDTA, 1.25M KCl) to each reaction and heating at 75°C for 2-3 minutes in heating blocks. Reactions were removed from the heat blocks and allowed to slowly cool to about 37°C. This was followed by addition of 23μl reverse transcription mix (20mM Tris-HCl pH 8, 10mM MgCl_2_, 0.1mg/ml Actinomycin D, 5mM DTT, 0.33mM dNTP) and 0.5μl M-MLV Reverse Transcriptase (Promega) per reaction and incubation at 37°C for 1 hour. Final reactions were precipitated, washed with ethanol, and placed in a speedvac for 5 minutes. DNA pellets were each resuspended in 10μl formamide with EDTA (1mM)/NaOH(0.1mM) (2:1) and heated to 95°C for 2-3 minutes followed by snap cooling on ice. Samples were electrophoresed through 8% polyacrylamide/TBE gels (SequaGel-8, National Diagnostics).

### Immobilized DNA recruitment assay

For transcription reactions using immobilized DNA templates, plasmids containing the *p21* promoter were first linearized by restriction enzyme cleavage with NotI, followed by cleavage with a second restriction enzyme, EcoRI, to generate sticky ends that were filled-in by Klenow DNAP with biotinylated dATP and dUTP. After removal of excess nucleotides, the biotinylated fragments were incubated with streptavidin-coated magnetic beads (Dynal, Invitrogen) and purified from un-biotinylated DNA using a magnet. The immobilized *p21* templates (250 fmoles) were each incubated with HeLa nuclear extract and reagents similar to *in vitro* transcription as previously reported [23]. Protein complexes were allowed to form a PIC on the promoter followed by treatment with the drug compounds. Transcription was initiated by adding NTPs and active transcription complexes were subsequently captured.

### Western blotting

Captured proteins from the recruitment assay were electrophoresed through 10% polyacrylamide/TBE gels and transferred to a nitrocellulose membrane. Immunoblots were probed using antibodies to P-Ser5 CTD (H14) and P-Ser2 CTD (H5) from Covance; and antibodies to RNAP II (sc-9001), TBP (sc-273), TFIIB (sc-225), and CDK9 (sc-8338) from Santa Cruz Biotechnologies.

## 


