# Supplementary material for: Identification of Kinase Inhibitors that Target Transcription Initiation by RNA Polymerase II
Source: Oncotarget. 2011 Jan 27;2(1-2):18–28. doi: 10.18632/oncotarget.212 (PMC3248145; doi:10.18632/oncotarget.212)
Supplement: Supplementary file 1 [file oncotarget-02-018-s001.pdf]

List of Kinase inhibitors screened.
BML-2832
http://www.enzolifesciences.com/BML-2832/kinase-inhibitor-library/

KINASE INHIBITORS	INTENDED TARGET		KINASE INHIBITORS	INTENDED TARGET
PD-98059	MEK		KN-93	CaMK II
U-0126	MEK		ML-7	MLCK
SB-203580	p38 MAPK		ML-9	MLCK
H-7	PKA, PKG, MLCK, and PKC.		2-Aminopurine	p58 PITSLRE beta1
H-9	PKA, PKG, MLCK, and PKC.		N9-Isopropyl-olomoucine	CDK
Staurosporine	Pan-specific		Olomoucine	CDK
AG-494	EGFRK, PDGFRK		iso-Olomoucine	Negative control for olomoucine.
AG-825	HER1-2		Roscovitine	CDK
Lavendustin A	EGFRK		5-Iodotubercidin	ERK2, adenosine kinase, CK1, CK2,
RG-14620	EGFRK		LFM-A13	BTK
Tyrphostin 23	EGFRK		SB-202190	p38 MAPK
Tyrphostin 25	EGFRK		PP2	Src family
Tyrphostin 46	EGFRK, PDGFRK		ZM 336372	cRAF
Tyrphostin 47	EGFRK		SU 4312	Flk1
Tyrphostin 51	EGFRK		AG-1296	PDGFRK
Tyrphostin 1	Negative control for tyrosine kinase inhibitors.		GW 5074	cRAF
Tyrphostin AG 1288	Tyrosine kinases		Palmitoyl-DL-carnitine Cl	PKC
Tyrphostin AG 1478	EGFRK		Rottlerin	PKC delta
Tyrphostin AG 1295	Tyrosine kinases		Genistein	Tyrosine Kinases
Tyrphostin 9	PDGFRK		Daidzein	Negative control for Genistein.
HNMPA (Hydroxy-2-naphthalenylmethylphosphonic acid)	IRK		Erbstatin analog	EGFRK
PKC-412	PKC inhibitor		Quercetin dihydrate	PI 3-K
Piceatannol	Syk		SU1498	Flk1
PP1	Src family		ZM 449829	JAK-3
AG-490	JAK-2		BAY 11-7082	IKK pathway
AG-126	IRAK		DRB (5,6-Dichloro-1-β-D-ribofuranosylbenzimidazole)	CK II
AG-370	PDGFRK		HBDDE (2,2',3,3',4,4'-Hexahydroxy-1,1'-biphenyl-6,6'-dimethanol dimethyl ether)	PKC alpha, PKC gamma
AG-879	NGFRK		SP 600125	JNK
LY 294002	PI 3-K		Indirubin	GSK-3beta, CDK5
Wortmannin	PI 3-K		Indirubin-3'-monoxime	GSK-3beta
GF 109203X	PKC		Y-27632	ROCK
Hypericin	PKC		Kenpaullone	GSK-3beta
Ro 31-8220	PKC		Terreic acid	BTK
Sphingosine	PKC		Triciribine	Akt signaling pathway
H-89	PKA		BML-257	Akt
H-8	PKA, PKG		SC-514	IKK2
HA-1004	PKA, PKG		BML-259	Cdk5/p25
HA-1077	PKA, PKG		Apigenin	CK-II
HDBA (2-Hydroxy-5-(2,5-dihydroxybenzylamino)benzoic acid)	EGFRK, CaMK II		BML-265 (Erlotinib analog)	EGFRK
KN-62	CaMK II		Rapamycin	mTOR

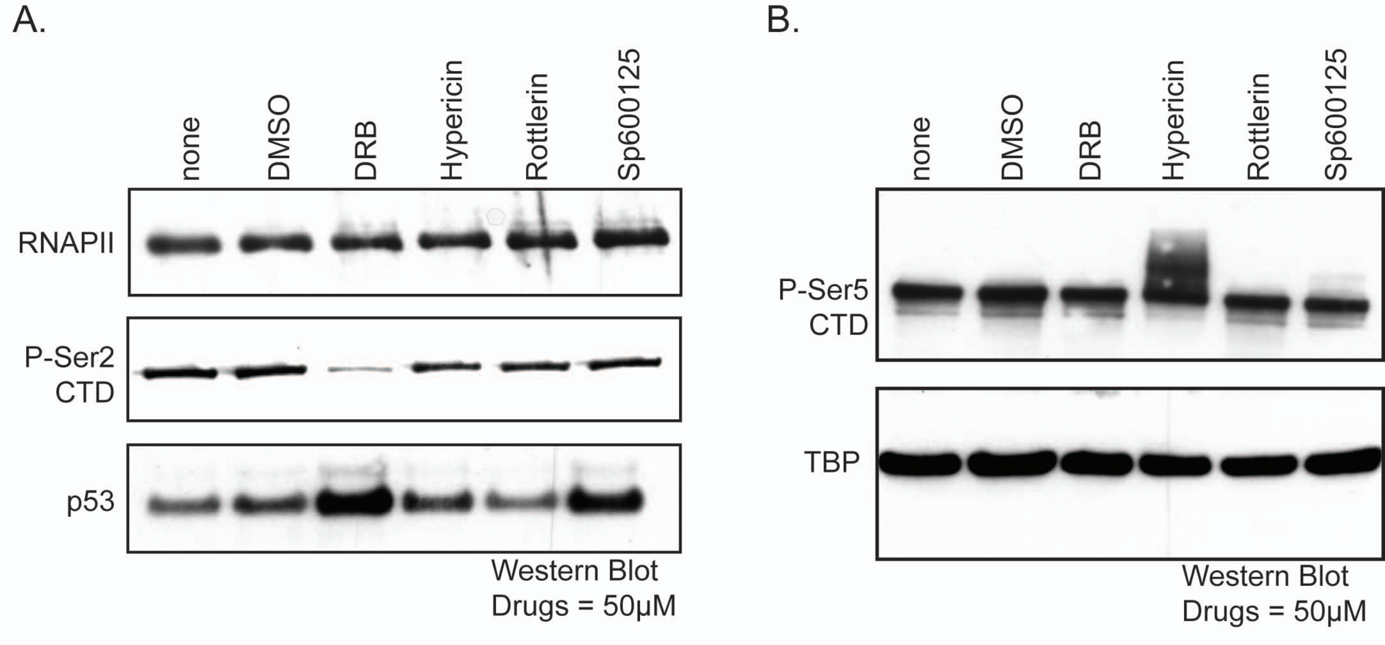
A.
none
DMSO
DRB
Hypericin
Rottlerin
Sp600125
RNAPII
P-Ser2 CTD
p53
Western Blot
Drugs = 50μM
B.
none
DMSO
DRB
Hypericin
Rottlerin
Sp600125
P-Ser5 CTD
TBP
Western Blot
Drugs = 50μM
